# Mining Hidden Knowledge About Illegal Compensation for Occupational Injury: Topic Model Approach

**DOI:** 10.2196/14763

**Published:** 2019-09-26

**Authors:** Jin-Young Min, Sung-Hee Song, HyeJin Kim, Kyoung-Bok Min

**Affiliations:** 1 Institute of Health and Environment Seoul National University Seoul Republic of Korea; 2 Department of Preventive Medicine Seoul National University College of Medicine Seoul Republic of Korea

**Keywords:** occupational injuries, worker’s compensation, social media, Korea

## Abstract

**Background:**

Although injured employees are legally covered by workers’ compensation insurance in South Korea, some employers make agreements to prevent the injured employees from claiming their compensation. Thus, this leads to underreporting of occupational injury statistics. Illegal compensation (called *gong-sang* in Korean) is a critical method used to underreport or cover-up occupational injuries. However, *gong-sang* is not counted in the official occupational injury statistics; therefore, we cannot identify *gong-sang*–related issues.

**Objective:**

This study aimed to analyze social media data using topic modeling to explore hidden knowledge about illegal compensation—*gong-sang*—for occupational injury in South Korea.

**Methods:**

We collected 2210 documents from social media data by filtering the keyword, *gong-sang*. The study period was between January 1, 2006, and December 31, 2017. After completing natural language processing of the Korean language, a morphological analyzer, we performed topic modeling using latent Dirichlet allocation (LDA) in the Python library, Gensim. A 10-topic model was selected and run with 3000 Gibbs sampling iterations to fit the model.

**Results:**

The LDA model was used to classify *gong-sang*–related documents into 4 categories from a total of 10 topics. Topic 1 was the greatest concern (60.5%). Workers who suffered from industrial accidents seemed to be worried about illegal compensation and legal insurance claims, wherein keywords on the choice between illegal compensation and legal insurance claims were included. In topic 2, keywords were associated with claims for industrial accident insurance benefits. Topics 3 and 4, as the second highest concern (19%), contained keywords implying the monetary compensation of *gong-sang*. Topics 5 to 10 included keywords on vulnerable jobs (ie, workers in the construction and defense industry, delivery riders, and foreign workers) and body parts (ie, injuries to the hands, face, teeth, lower limbs, and back) to *gong-sang*.

**Conclusions:**

We explored hidden knowledge to identify the salient issues surrounding *gong-sang* using the LDA model. These topics may provide valuable information to ensure the more efficient operation of South Korea’s occupational health and safety administration and protect vulnerable workers from illegal *gong-sang* compensation practices.

## Introduction

### Background

Occupational injuries, defined as work-related injuries, diseases, and death, are an important public health issue. They are one of the main causes of workers’ morbidity, disability, and mortality as well as substantial losses in social and economic activities. According to the International Labor Office (ILO), 2.3 million workers die from an occupational injury or a disease annually [1]. The global burden of occupational injuries has reached 4% of the global gross domestic product (approximately US $3 trillion) [1].

Although it is difficult to compare national rates of occupational injuries because of variations in legal and compensation criteria, South Korea’s occupational injury statistics have certain unique features, including the lowest nonfatal occupational injury rate alongside the highest death rate [2]. When compared with other Organization for Economic Co-operation and Development (OECD) member countries in 2014, South Korea’s nonfatal occupational injury rate of 0.53% was far below the OECD average of 2.7%, whereas fatal work-related deaths in the country were ranked the highest (ie, 10.8 per 100,000 people) [3]. South Korea also reported lower numbers of nonfatal occupational injuries and higher rates of fatal occupational injuries than European countries [2].

Workers in Korea are legally covered by workers’ compensation insurance when they receive more than 3 days of medical treatment [4]. However, some employers make agreements with workers to prevent them from applying for the compensation insurance benefit, even in cases requiring up to 4 days of treatment. Such agreements giving way for illegal compensation (*gong-sang* in Korean) is considered a critical example of occupational injury cover-up. Literally, “*gong-sang*” means a wound caused while performing official duties; in practice, it means an agreement between an employer and employee not covered by the worker’s compensation insurance where the employer pays directly for the worker’s compensation for medical treatment and suspension of employment when injured at work. It is unfortunate that illegal compensation or *gong-sang* rates are not captured by official occupational injury statistics, and, thus, it is impossible to monitor illegally compensated occupational injuries using the conventional system [5,6].

In this era of digital information and communication technologies, many people post their reviews of products and services from restaurants, hotels, and hospitals on the Web. They also seek professional advice on health and legal issues through social media websites. In these circumstances, seeking advice about illegal compensation or *gong-sang* may be similar. Injured workers who are forced by employers to agree to illegal compensation may discuss it with experts, experienced people, and the public using social media. If this is the case, Web-based data may be useful for identifying the undisclosed contents of *gong-sang* provided for injured employees and the hidden administration of occupational health and safety.

### Objectives

This study aimed to analyze social media data using topic modeling to explore issues surrounding *gong-sang*. Topic modeling is a widely used text mining approach for analyzing large volumes of unlabeled documents to discover hidden textual patterns [7]. Specific concerns addressed when analyzing data about *gong-sang* included the key issues described by the victims: what type of worker is vulnerable, and what kind of injuries are subject to illegal compensation.

## Methods

### Data Extraction and Processing

We collected social media data from knowledge-sharing websites, such as Naver Knowledge In. Knowledge-sharing websites allow people to interact with each other and share their knowledge by asking and answering questions. These websites have an accumulated knowledge database through a question-answering system. From the database, this study focused on posts pertaining to occupational injury and responded through a certified labor attorney as expert counseling. Web scraping was used to scrape 374,308 documents with the keyword, *occupational injury*. Using the keyword, *gong-sang*, the data were filtered, and 3692 documents were identified in the social media context. We further removed 1231 duplicated documents and applied a limited study period between January 1, 2006, and December 31, 2017. Finally, 2210 documents were included for further analysis. We analyzed Google Trends data to highlight public attention to *gong-sang* issues and displayed the trend for search queries on occupational accidents, *gong-sang* handling, and workers’ compensation. Google Trends provided a time series index of the number of the queries entered into Google for a given topic in South Korea across 12 years (2006-2017). The value displayed in Google Trends is not based on the total number of searches but represents the search interest relative to the highest point on the chart for the given time and geographic region.

Social media posts pertaining to occupational injury were processed to transform unstructured textual documents into structured data using the Python package. For natural language processing of the Korean language, KoNLPy, a relatively new open source morphological analyzer library, developed by Park and Cho [8], was used. Thereafter, unnecessary sentence components (ie, special characteristics, numbers, and punctuations) and meaningless words (ie, *a*, *the*, and *it*) in the text file were removed, and nouns were extracted with more than 2 letters. Next, a term-document matrix was constructed, which used term frequency–inverse document frequency (TF-IDF) weights for information retrieval. A TF-IDF algorithm evaluates how important a word is in a document in a collection or corpus, with the value increasing proportionally to the number of times a word appears in a document [9]. To provide relationships between the keywords in the *gong-sang*-related documents, we analyzed co-occurrence network of high-frequency words using Gephi modules in Python.

### Applying Topic Modeling

Topic modeling is an emerging field in machine learning that detects the hidden topics in large textual corpora. Latent Dirichlet allocation (LDA) is one of the most popular topic modeling techniques. LDA states that each document in a corpus is a mixture of latent topics and that each word’s presence is attributable to one of the document’s topics [7]. In the LDA model, topic distribution over each document and word distribution over each topic share the common Dirichlet prior [7]. We used LDA in Gensim, a Python library, for topic modeling. Perplexity was evaluated to determine the optimal number of topics and then computed to determine the difference in perplexity change. Perplexity is a common method to measure how well a probability distribution predicts a held-out sample [7]. A lower value of the difference in perplexity change denotes a better probabilistic model. LDA defines a *topic* as a probability distribution over a fixed vocabulary in a given document [7]. The parameter λ determines the weight given to the probability of a term within a topic relative to its lift. Setting λ=1 results in the familiar ranking of terms in decreasing order of their topic-specific probability, whereas setting λ=0 ranks terms solely by their lift. We set λ=1 and run the LDA with 3000 Gibbs sampling iterations. A 10-topic model with the lowest difference in perplexity change was used, and topics were plotted using circles on a 2-dimensional plane along the transverse (PC1) and longitudinal (PC2) axes. In this visualization, each topic was presented as a circle, and the circle area represented the prevalence of each topic. The centers of each topic were determined by computing the distance between topics. Furthermore, we used multidimensional scaling to represent the intertopic distances in 2 dimensions [10].

## Results

### Summary Statistics

**Figure 1:**
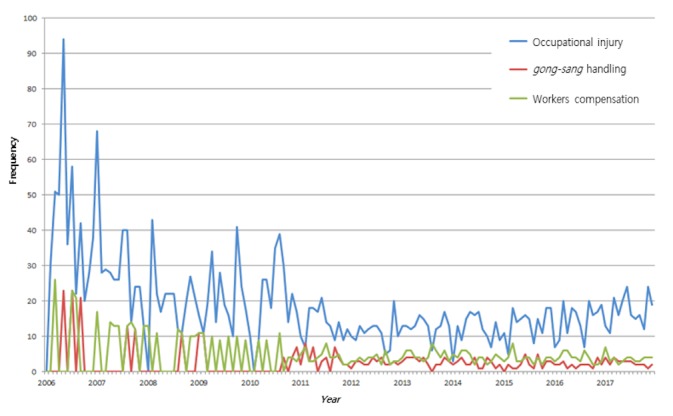
The trend on Google Trends for search queries on gong-sang–related topics—occupational accident, gong-sang handling, and workers’ compensation—between 2006 and 2017.

Figure 1 displays the trends on Google Trends for search queries on *gong-sang*–related topics, specifically occupational accidents, *gong-sang* handling, and workers’ compensation, over 12 years. Among them, occupational accidents was the most popular term. The popularity of occupational accidents nonlinearly decreased from 2006 until 2012; subsequently, it steadily increased. In the queries for *gong-sang* handling and workers’ compensation, although there was a wide fluctuation in their popularity between 2006 and 2010, the queries’ concern continued even when the popularity was low, relative to occupational accidents.

The value is calculated relative to the highest point on the chart for 12 years in South Korea: a value of 100 is the highest popularity of each term, and a value of 50 means that these terms were searched as frequently as half of the highest popularity.

We identified 2210 *gong-sang*–related documents from the expert counseling service between January 1, 2006, and December 31, 2017. Table 1 shows the distribution of the number of documents during the study period. The number of documents was less than 100 in 2006 and 2007; however, over the years, there has been a gradual increase in the documents.

**Figure 2:**
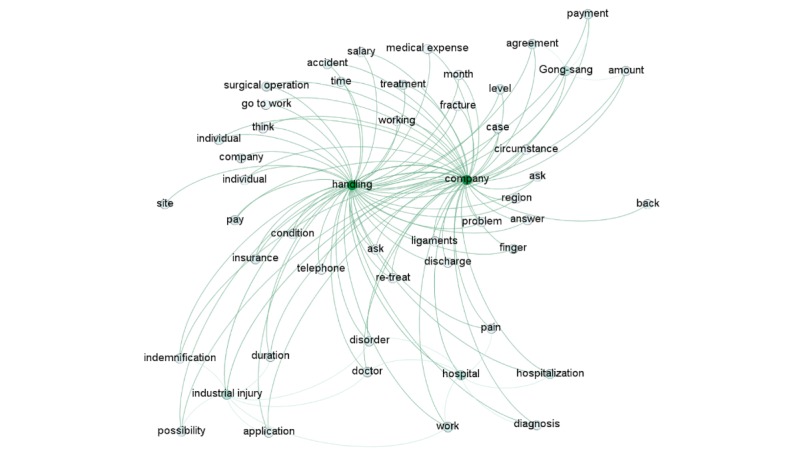
Co-occurrence network of high-frequency words in the gong-sang–related documents.

Figure 2 shows a word cloud display, a visual representation of the word frequency within the *gong-sang*–related documentation. The clouds provide greater prominence to words that appear more frequently in the given text.

Figure 2 shows co-occurrence network of high frequency words in the *gong-sang*-related documents. A node represents the co-occurrence relationship between two words appearing in the same article simultaneously. Nodes with a large degree are considered as high-connectivity nodes or hub nodes.

**Table 1:** The number of gong-sang-related documents between 2006 and 2017 (N=2210).

Year	Number of documents, n (%)
2006	49 (2.2)
2007	70 (3.2)
2008	110 (5.0)
2009	156 (7.1)
2010	111 (5.0)
2011	121 (5.5)
2012	156 (7.1)
2013	197 (8.9)
2014	236 (10.7)
2015	289 (13.1)
2016	329 (14.9)
2017	386 (17.5)

### Topic Modeling

In Table 2, the top 20 most frequent words have been provided. Intuitive words about *gong-sang* (ie, handling, company, *gong-sang*, and occupational injury) were sorted into the upper half. Words such as hospital, treatment, level, surgical operation, hospitalization, accident, site, diagnosis, and back were associated with bodily injury.

**Figure 3:**
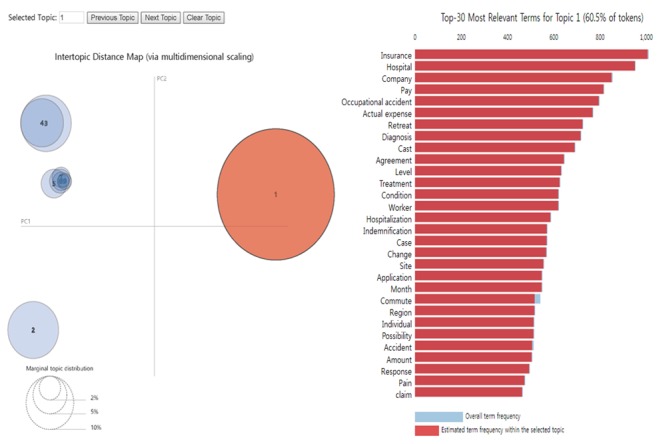
The layout of latent Dirichlet allocation of the gong-sang–related documents, with a global topic view on the left and the term bar charts on the right. PC1: transverse axe; PC2: longitudinal axe.

To classify the given documents on a particular topic, we determined the number of topics by calculating their perplexity. We adopted a 10-topic model with the lowest difference in perplexity changes and plotted the topics as seen in Figure 3. The overall view of topics is expressed as circles on the left panel, and the top 20 most useful terms for interpreting each topic are shown in a bar chart on the right panel.

**Table 2:** Top 20 high-frequency words in the gong-sang–related documents.

Keywords	Frequency
handling	6076
company	5251
gong-sang	4188
occupational injury	3682
hospital	3238
treatment	2371
level	1684
surgical operation	1501
insurance	1242
hospitalization	1239
accident	1032
pay	1022
case	970
site	929
condition	911
agreement	868
re-treat	837
diagnosis	822
working	791
back	750

Keywords on each topic with a percentage of the given documents are summarized in Table 3. Topic 1 was the most popular at 60.5%. We interpreted this topic as the choice between illegal compensation (*gong-sang*) and legal insurance claims (actual medical cost insurance as private insurance or industrial accident compensation insurance as social insurance). Topic 2 included keywords associated with claims for industrial accident insurance benefits. Topics 3 and 4 were classified as similar subjects: monetary compensation for subcontractors (topic 3) and daily workers (topic 4). Approximately, 11% corresponded to topics 5 to 10. These 5 topics involved keywords relating to injured body parts and the employment status of *gong-sang*. The words included hand injury (topic 5), construction workers (topic 6), accidental injury to the body (topic 7), vulnerable job (topic 8), lower limb and back injury (topic 9), and foreign workers (topic 10).

**Table 3:** Topic classification and keywords on gong-sang.

Classification and topic name		Keywords	Values, %
**Choice between illegal compensation and legal insurance claims**			
	Topic 1: Actual medical cost insurance or industrial accident compensation insurance	insurance, hospital, company, pay, occupational accident, actual expense, retreat, diagnosis, agreement, treatment, hospitalization, indemnification, application, amount, claim	60.5
**Claim for industrial accident insurance benefits**			
	Topic 2: Industrial accident insurance benefits	payment, surgical operation, medical expenses, fracture, burden, employee, medical treatment, allowance, receipt, disability, salary, bonus, guarantee, public corporation, business owner	11.3
**Monetary compensation for subcontractors and daily workers**			
	Topic 3: Money compensation for subcontractors	salary, subcontract, hammering, claim, calculation, date, annual leave, medical expenses, scar, in-company, lumbar, basic pay, shipyard, money, industrial accident	11.1
	Topic 4: Money compensation for daily workers	muscle, X-ray, daily pay, daily wage, action, total amount, loss, disability, injury, hospital charge, convalescence, work, business owner, refusal, exemption	8.1
**Descriptions of illegal compensation: vulnerable body part and employment status**			
	Topic 5: Hand injuries	Finger, record, general practice, needle, suture, first medical examination, right hand, stitches, materials, operation, thumb, index finger, emergency department, centimeter, laceration	2.9
	Topic 6: Construction workers	medical certificate, severance pay, progression, region, subcontract, last year, Saturday, duration, reason, disk, resign, one’s own expense, building completion, official vacation, flange	2.0
	Topic 7: Accidental injury to the body	day labor, face, circumstance, acquaintance, tooth fracture, degeneration, traffic accident, cause, dental crown, exposure, nitric acid, breathing, right, chest, implant	2.7
	Topic 8: Vulnerable jobs	metal pin, claim, construction, guard, carpenter, pickup, delivery, outskirts, penalty, defense industry, rider, memorandum, separate collection, defense personnel, McDonalds	1.7
	Topic 9: Lower limb and back injuries	knee, cartilage, cast, cruciate ligaments, height, mediation, ligaments, rupture, compensation, coin patch, defeat, medicine, back, tarsal bone, technician	0.9
	Topic 10: Foreign workers	outplacement, inflammation, evidence, patient, foreigner, recruitment, rotation, overwork, hospitalization, surgical operation, trauma, South Korea, Hangeul, (Korean alphabet), reentry, false	0.6

## Discussion

### Principal Findings

Illegal compensation (ie, *gong-sang*) because of occupational injury is a serious social problem in South Korea. At the company level, *gong-sang* entails a violation of an employer’s legal obligations, for example, the obligation to declare serious industrial accidents. However, as *gong-sang* is used to avoid penalties, including court proceedings under the Korean Industrial Safety and Health Act, avoid increases in insurance premiums, and, in case of construction companies, avoid restrictions in government-ordered construction projects, companies often force injured workers to agree to *gong-sang*. At the public level, *gong-sang* could be a financial burden for the National Health Insurance scheme, because workers’ injuries that are not officially reported as industrial accidents will be covered by the National Health Insurance and not the industrial accident compensation insurance. The practice of *gong-sang* in the workplace consequently leads to the distortion of official occupational accident statistics.

Despite the significance of *gong-sang*, it is not formally declared. Some surveys have provided a limited understanding of *gong-sang* by focusing on workers in a certain job. Our study analyzed a Web-based knowledge search dataset from 2006 to 2017 and identified the major issues surrounding *gong-sang*. The results of topic modeling were classified into 4 categories from 10 topics. Topic 1 was of the greatest concern (60.5%). Workers who suffered from industrial accidents seemed to be worried about illegal compensation and legal insurance claims. There were words alluding to *gong-sang*, such as company, occupational accident, agreement, diagnosis, and indemnification. Some words implied legal compensation: actual medical cost insurance (ie, hospital, actual expense, hospitalization, treatment, and application) as personal insurance and workers’ compensation insurance (ie, insurance, pay, retreat, amount, and claim) as social insurance. According to a study of industrial accidents [6,11], injured workers (those requiring medical care for more than 3 days) often tacitly agreed to *gong-sang* with the employer. Workers compensated with *gong-sang* were often in trouble because of insufficient company payouts and the aftereffects of their occupational accident. Thereafter, the entire burden would be on the individual. Such circumstances may lead workers to be concerned about whether they were receiving illegal compensation (*gong-sang*) or legal insurance claims.

The next highest concern (19.2%) was the *gong-sang* monetary compensation, and topics 3 and 4 corresponded to this. There were keywords estimating monetary rewards from occupational injuries (ie, salary, claim, calculation, date, annual leave, medical expense, scar, lumbar, basic pay, money, industrial accident, muscle, X-ray, total amount, disability, injury, hospital charge, and convalescence) and conjecturing the company’s attitude (ie, loss, business owners, action, refusal, and exemption). Vulnerable workers, such as subcontractors (ie, subcontract, hammering, shipyard, and in-company) and daily workers (ie, daily pay, daily wage, and work), seemed to be more involved in this issue. A subcontractor is hired by a general contractor to perform a specific task as part of an overall project. Subcontractors or daily workers, as a representative class excluded from social insurance, were often forced to accept *gong-sang* from company or business owners in the event of an occupational accident and were known to prefer it often [6,12].

Another important classification was claiming for industrial accident insurance benefits. Industrial accident compensation benefit is a social insurance system administered by the state to promptly compensate workers who have suffered an industrial accident and to relieve the employer’s temporary economic burden. Topic 2 included keywords, implying medical care benefits (ie, payment, surgical operation, medical expenses, fracture, medical treatment, and receipt), unemployment benefits (ie, allowance, salary, bonus, guarantee, and business owner), and disability benefits (ie, burden, employee, disability, and public corporation), which are components of industrial accident compensation benefits.

The remaining topics (topics 5-10) were classified as descriptions of illegal compensation, focusing on vulnerable body parts and employment status. The main keywords of topic 5 referred to hand injuries (ie, finger, general practice, needle, suture, first medical examination, right hand, stiches, surgical operation, thumb, index finger, emergency department, centimeter, and laceration). Topic 9 suggested keywords corresponding to the lower limbs (ie, knee, cartilage, cast, cruciate ligaments, ligaments, rupture, and tarsal bone) and back injuries (ie, coin patch, medicine, and back). In topic 7, keywords also suggested bodily injuries (ie, face, tooth fracture, degeneration, dental crown, breathing, chest, and implant) because of occupational accidents (ie, day labor, traffic accident, cause, exposure, and nitric acid). Despite the lack of official statistics, it seemed that fatal industrial accidents were covered by workers’ compensation insurance, and *gong-sang* was taken for granted in nonfatal injury cases. Our data indicated that nonfatal injuries that occurred to the hands, face, teeth, lower limbs, and back were often associated with *gong-sang*.

The employment status vulnerable to *gong-sang* seemed to be referenced most in topics 6, 8, and 10. There were keywords such as construction workers (ie, region, subcontract, Saturday, duration, building completion, and flange) in topic 6, vulnerable jobs (ie, construction, guard, carpenter, pickup, delivery, defense industry, rider, separate collection, defense personnel, and McDonalds) in topic 8, and foreign workers (ie, foreigner, recruitment, rotation, overwork, South Korea, Hangeul, and reentry) in topic 10. Additional words across these 3 topics were likely to refer to managing *gong-sang*. For example, the type of compensation (ie, medical certificate, severance pay, progression, last year, reason, disk, resign, one’s own expense, and official vacation in topic 6) and the consequences (ie, outplacement, inflammation, evidence, patient, hospitalization, surgical operation, trauma, and false in topic 10). Workers in precarious jobs, such as builders, guards, and delivery persons, lack occupational health and safety protection and social security coverage [13]. Although they are covered by workers’ compensation insurance, workers in precarious jobs tend to prefer *gong-sang* because they are afraid of the disadvantages related to their work due to official insurance claims [6,12]. Our results indicated that workers in defense-related industries (or defense personnel) and foreign workers were particularly vulnerable. A worker in the defense industry refers to young men who are treated as an exception and have their military duties substituted within a fixed duration. Although workers in defense-related industries are treated unfairly and are offered *gong-sang* in the case of occupational injury, they tend to be overlooked because of the mandatory replacement period for military service [14]. Meanwhile, foreign workers have the same basic labor rights as Korean nationals. Nonetheless, many foreign workers remain unaware of the industrial health and safety provisions in different countries, and their job stability tends to be poor because of their illegal immigration status [15,16]; therefore, they are not offered appropriate compensation in the workplace.

### Implication

This is the first study to use topic modeling to analyze unstructured Web-based text data about *gong-sang*–related topics. Our study provides important insights into the actual circumferences surrounding *gong-sang*, for example, injured workers’ concerns (as seen in topics 1-4) about *gong-sang* and the types of jobs and injuries associated with *gong-sang* (topics 5-10). However, illegal compensation or *gong-sang* is considered as a situation exclusive to South Korea. According to our observations, companies would like to limit their penalties (such as increases in insurance premiums and restrictions in government-ordered construction projects) derived from employees’ injury or illness and impose illegal compensations for injured workers. However, it is not known whether regulations and/or insurance in other countries obligate employers to compensate injured workers. For example, some international firms have arrangements wherein they offer a pickup and drop-off service for workers who cannot walk. Regulations in the West allow such services and companies to not register these people as temporarily unemployed when they conduct adapted tasks. Eventually, illegal workers’ compensation in South Korea may not be considered as a crime or fraud in the rest of the world. The interpretation and application of our results should be executed cautiously.

### Limitation

This study needs to address the drawbacks of topic modeling. The topic modeling technique is highly effective for extracting knowledge from previously unknown information contained in unstructured big data [17,18] and has been widely used in the field of biological and medical document mining. Nonetheless, as is the case with all text mining approaches, difficulties arise when making interpretations and subjective validations, as the *truth* contained in the given documents and the number of relevant themes are not known *a priori* [18]. We determined the best topic model by applying 3000 iterative processes and a perplexity-based method. However, the total number of topics remains unknown and depends on reasonable deductions. Future study is required to validate our perspective of *gong-sang–*related issues. A comparative study of another methodological approach (ie, grounded theory and deep learning) could be useful for knowledge discovery and comprehension.

### Conclusions

In conclusion, we explored unstructured Web-based data and discovered hidden knowledge to identify the salient issues surrounding *gong-sang*. The topics formulated by LDA topic modeling included queries about legal insurance claims, such as private or social insurance (topics 1-2), monetary compensation (topics 3-4), injured body parts (topics 5, 7, and 9), and the type of jobs (topics 6, 8, and 10) vulnerable to *gong-sang*. These topics may provide valuable information to ensure further efficient operation of South Korea’s occupational health and safety administration and protect vulnerable workers from illegal *gong-sang* compensation practices.

## Abbreviations

ILO: International Labor Office
LDA: latent Dirichlet allocation
OECD: Organization for Economic Co-operation and Development
TF-IDF: term frequency-inverse document frequency
